# Endoscopic diagnosis and management of biliary neuroendocrine tumor with prior Roux-en-Y choledochojejunostomy: a case report

**DOI:** 10.1016/j.igie.2026.03.017

**Published:** 2026-04-01

**Authors:** Alec Hope, Hasib Ahmadzai, Philip Ian Craig

**Affiliations:** 1 Department of Gastroenterology and Hepatology, Westmead Hospital, Sydney, New South Wales, Australia; 2 Department of Gastroenterology and Hepatology, Blacktown Hospital, Sydney, New South Wales, Australia; 3 Department of Gastroenterology and Hepatology, St George Hospital, Sydney, New South Wales, Australia; 4 St George Hospital Clinical School, University of New South Wales, Sydney, New South Wales, Australia

## Abstract

A 74-year-old woman with a previous Roux-en-Y choledochojejunostomy presented with biliary sepsis, *Escherichia coli* bacteremia, and a left-sided intrahepatic biliary stricture. Initial management included percutaneous transhepatic cholangiography with external biliary drainage. Colonoscope-directed cholangioscopy enabled biopsies and balloon dilatation because of the colonoscope's suitable length and channel diameter. Histology revealed a poorly differentiated neuroendocrine tumor of the bile duct. Surgical resection was abandoned intraoperatively because of peritoneal metastases. Following chemotherapy, the patient re-presented with biliary sepsis. Endoscopic retrograde cholangiography using a pediatric colonoscope allowed placement of an uncovered self-expandable metal stent across the intrahepatic stricture. The patient underwent lutetium therapy and was clinically stable at 12-month follow-up. This case highlights biliary neuroendocrine tumor as a rare cause of stricture and demonstrates the utility of colonoscope-directed cholangioscopy and endoscopic retrograde cholangiography for diagnosis and management.

## Introduction

The diagnosis and management of biliary sepsis are complicated in patients with previous hepatobiliary surgery including Roux-en-Y choledochojejunostomy.^1^^,^^2^ The length of the alimentary and biliary limbs usually precludes the use of a conventional duodenoscope, whereas cholangioscopy requires a larger channel than that provided by conventional enteroscopes. Colonoscope-directed procedures may provide adequate scope length and channel diameter to facilitate biliary tree assessment and intervention.

## Case description

A 74-year-old woman reported a history of complex choledocholithiasis not amenable to retrieval during open cholecystectomy and bile duct exploration 46 years earlier. Endoscopic retrograde cholangiography (ERC) was not available; therefore, Roux-en-Y choledochojejunostomy was performed. Then, 10 years ago the patient underwent enteroscope-directed ERC to treat cholangitis and choledocholithiasis. Other medical history included idiopathic pulmonary fibrosis, hypertension, hypercholesterolemia, and gastroesophageal reflux disease. There was no significant family history. She was a nonsmoker and consumed 2 standard units of alcohol up to 3 times a week.

The patient presented with abdominal pain and fever. Examination revealed tachycardia, fever, and right upper quadrant tenderness. Laboratory studies revealed leukocytosis, elevated C-reactive protein at 108 mg/L (<3 mg/L), and deranged liver enzymes in a cholestatic pattern with normal serum bilirubin of 0.58 mg/dL (<1.17 mg/dL). Computed tomography (CT) of the abdomen showed a rim-enhancing hypodense lesion in segments 2 and 3 of the liver with associated localized left-sided biliary obstruction (Fig. 1). As timely biliary decompression was the priority and given the anticipated procedural complexity because of altered anatomy, an 8.5F percutaneous biliary drain was placed across a left-sided intrahepatic stricture (Fig. 2). Blood cultures grew extended-spectrum beta-lactamase (ESBL)–producing *Escherichia coli (E coli),* necessitating treatment with intravenous meropenem for 2 weeks.

**Figure 1:**
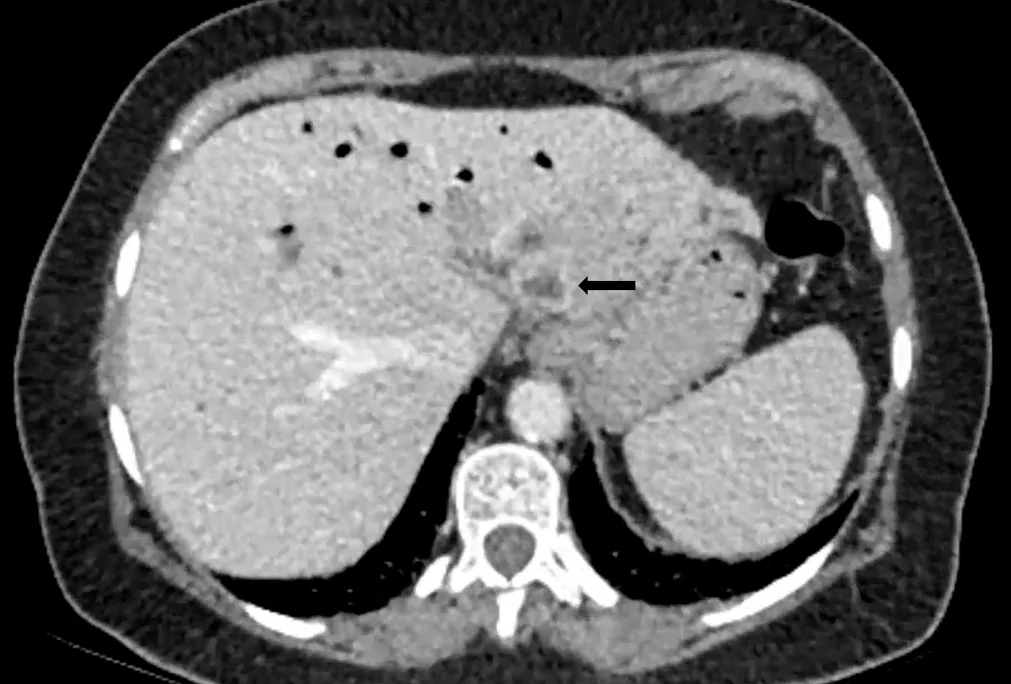
Computed tomography scan showing a left-sided hepatic abscess marked with a *black arrow*.

**Figure 2:**
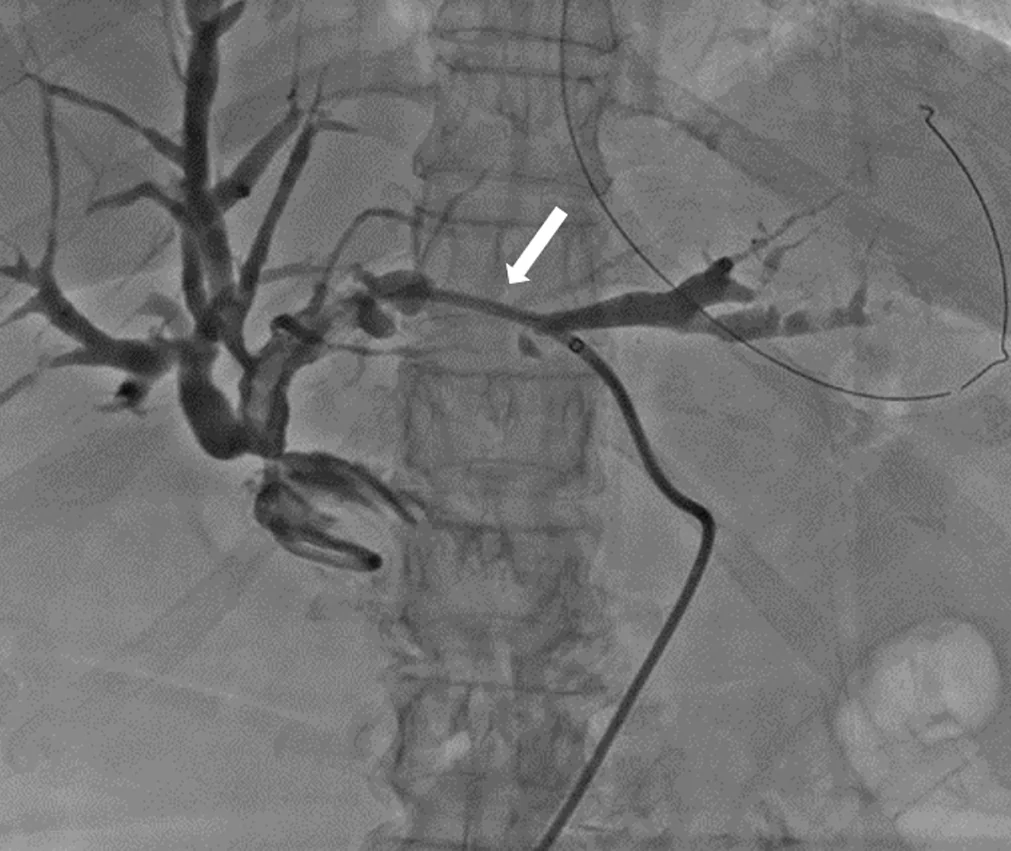
Fluoroscopic image of percutaneous biliary drain across the left peripheral biliary stricture marked with a *white arrow*.

Two weeks after external biliary drain placement, colonoscope-directed rendezvous ERC was performed to further assess the biliary stricture. An adult colonoscope (CF-HQ190, Olympus, Center Valley, Pa, USA) was used to access the biliary-enteric anastomosis (Video 1, available online at www.igiejournal.org). This provided a 3.7-mm channel to accommodate passage of a 3.3-mm cholangioscope (SpyGlass DS Direct Visualization System, Boston Scientific, Marlborough, Mass, USA). A 0.025-inch guidewire (VisiGlide, Olympus) was fed down the percutaneous biliary drain and captured with a snare before the cholangioscope was back fed over the guidewire into the biliary tree through the colonoscope. Cholangioscopic assessment of the left-sided biliary tree showed a peripheral indeterminate biliary stricture with certain suspicious features including a mass encroaching into the lumen, dark color changes, and neovascularization (Fig. 3). Cholangioscopic forceps (SpyBite, Boston Scientific) were used to obtain biopsy specimens, and then the stricture subsequently dilated to 8 mm (CRE balloon dilator, Boston Scientific). Because there was then excellent flow of contrast into the jejunum, we elected to remove the external biliary drain. There were no procedural adverse events, and ongoing clinical improvement enabled discharge with a short course of oral antibiotics.

**Figure 3:**
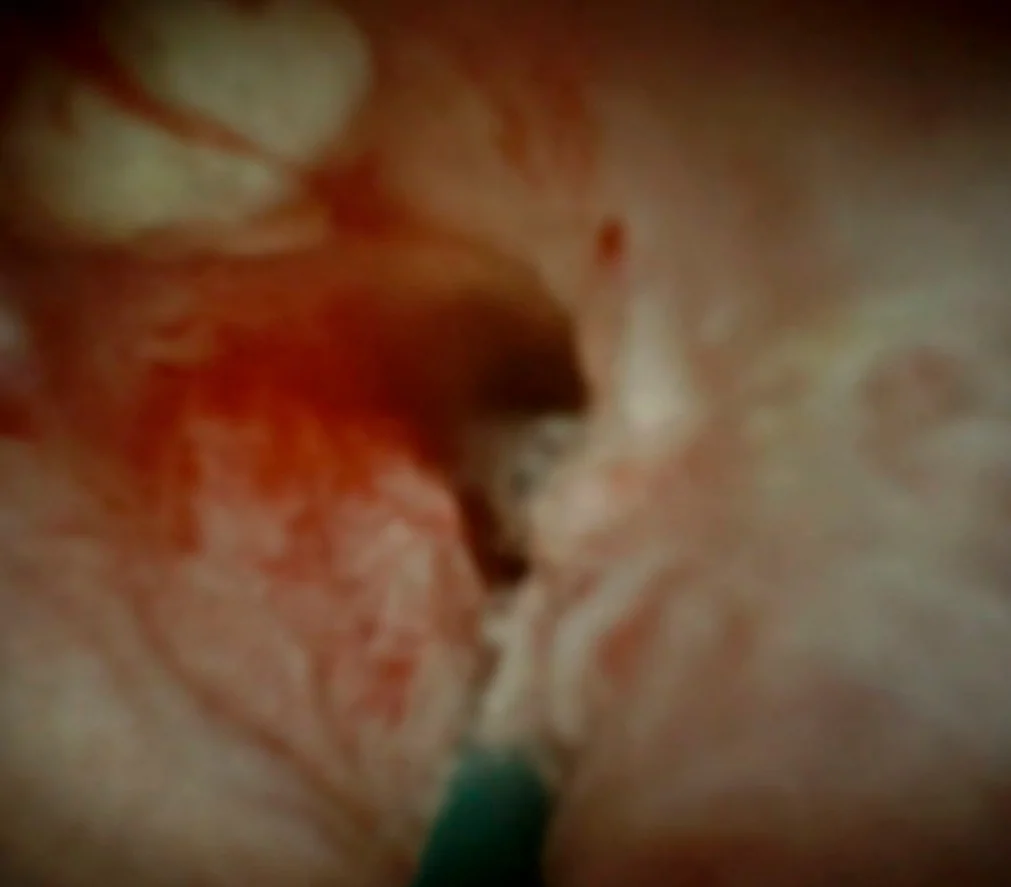
Cholangioscopic image of the biliary stricture.

Histopathological assessment showed a poorly differentiated biliary neuroendocrine tumor (Fig. 4) with classic staining characteristics including positive chromogranin A and cluster of differentiation 56. Antigen Kiel 67 was high at 60% to 70%, indicative of poor differentiation. In this case, both fluorodeoxyglucose and 1,4,7,10-tetraazacyclododecane-1,4,7,10-tetraacetic acid-octreotate (DotaTATE) positron emission tomography scans showed no evidence of metastatic disease spread. Open liver resection was attempted; however, metastatic disease was identified in the peritoneum. The patient was treated with 3 cycles of carboplatin and etoposide-based chemotherapy. She tolerated therapy well, although she noted fatigue and nausea as prominent adverse effects.

**Figure 4:**
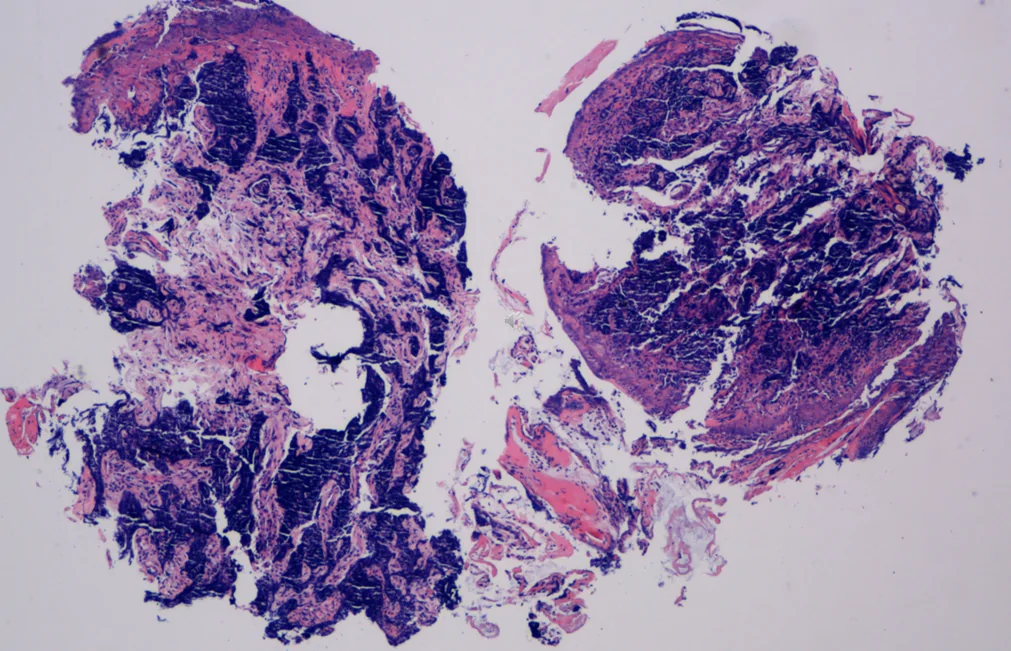
Biliary neuroendocrine tumor (hemotoxylin and eosin, original magnification x40, staining nuclei *purple* and cytoplasm *pink*).

Nine months after her initial presentation, the patient re-presented with recurrent cholangitis (Fig. 5). Blood cultures again grew ESBL-producing *E coli*, treated with intravenous meropenem. Abdominal CT scan demonstrated persistent dilatation of the left-sided peripheral biliary ducts in segments 2 and 3. We elected to perform a repeat ERC to stent the biliary obstruction using a pediatric colonoscope (PCF-H190, Olympus) with a 3.2-mm biopsy channel (Video 2, available online at www.igiejournal.org). A guidewire and catheter were passed across the stricture into the peripheral left-sided ducts with contrast injection confirming the stricture, which was dilated with an 8-mm balloon (CRE, Boston Scientific). An 8-mm × 60-mm biliary self-expandable uncovered metal stent (Epic, Boston Scientific) was positioned across the stricture with prompt drainage of contrast (Fig. 6). There were no procedural adverse events. The patient was discharged with ongoing improvement in her liver enzymes with subsequent normalization of serum albumin concentration and C-reactive protein levels. The patient underwent 3 cycles of treatment with lutetium-DotaTATE and remained well at 12-month follow-up.

**Figure 5:**
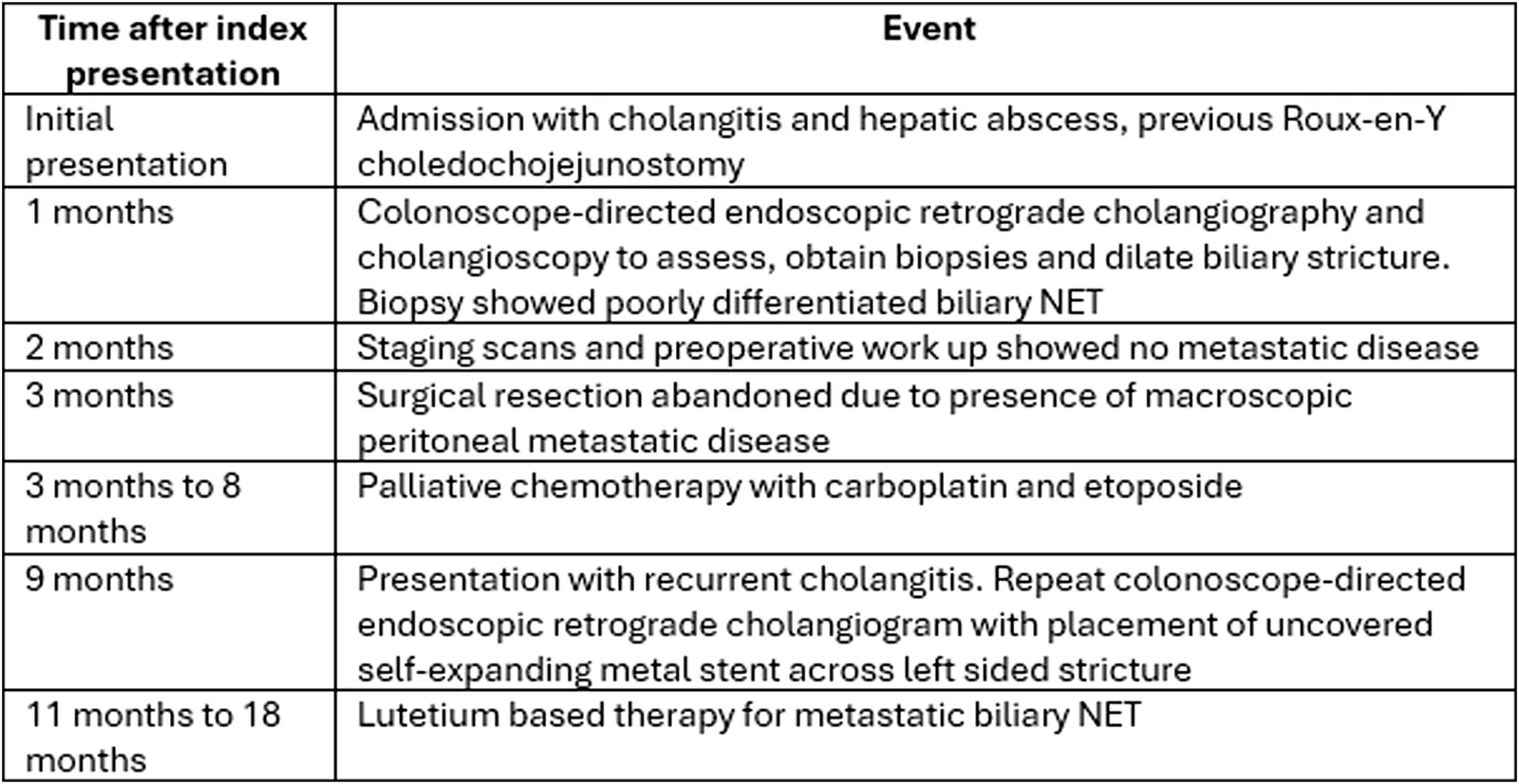
Clinical timeline. *NET*, Neuroendocrine tumor.

**Figure 6:**
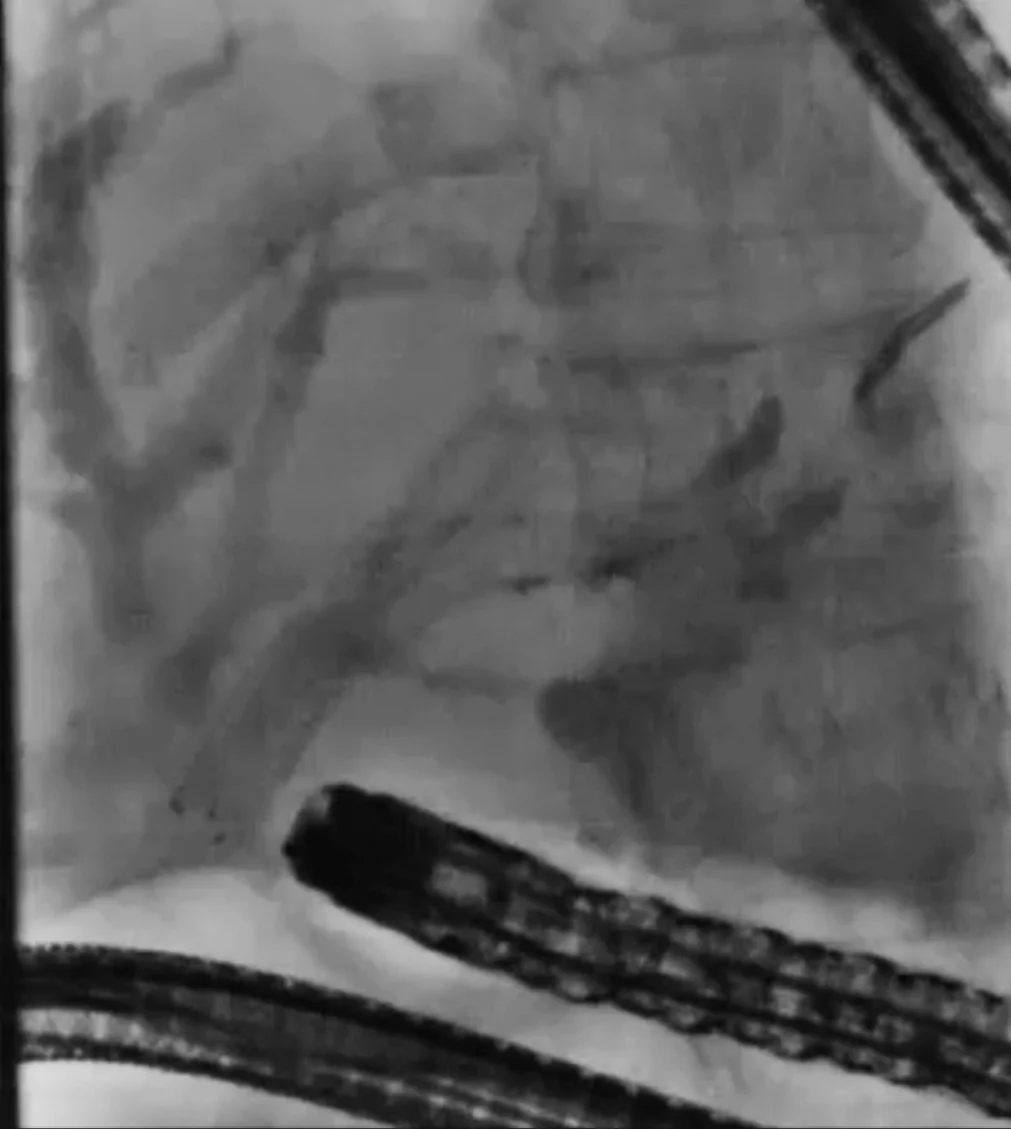
Fluoroscopic image of intrahepatic metal stent placement.

## Discussion

This case highlights the innovative use of colonoscope-directed cholangioscopy and ERC in a patient with Roux-en-Y choledochojejunostomy and biliary sepsis. This approach facilitated visual inspection of the indeterminate biliary stricture and targeted biopsies leading to the diagnosis of a rare biliary tumor. The strengths of this approach include utilization of a multidisciplinary team approach to management, a tailored endoscopic strategy, and the successful assessment, biopsy, and stenting of the biliary stricture despite altered anatomy.

Endoscopic ultrasound (EUS)-guided procedures including hepaticogastrostomy and EUS-assisted ERC are alternative procedures that allow therapeutic drainage of the bile duct, particularly when the enterobiliary anastomosis is inaccessible, necessitating transgastric approaches.^3^ Although EUS was considered to achieve internal biliary drainage, the literature demonstrates colonoscope-assisted ERCP and cholangioscopy as a safe and efficacious approach in patients with surgically altered biliary anatomy.^4^, ^5^, ^6^, ^7^

Limitations include an initial delay in diagnosis because of anatomical complexity and relative rarity of biliary NETs contributing to initial diagnostic uncertainty. NETs of the biliary tree are uncommon tumors arising from neuroendocrine cells. Functional tumors can secrete hormones that cause symptoms such as flushing and diarrhea, while others are nonfunctional. The etiology of biliary NETs is not well understood but believed to be related to chronic inflammation inducing metaplasia leading to malignancy. Biliary NETs are rare causes of biliary strictures and account for only 0.2% to 2% of all gastrointestinal NETs.^8^ They are typically diagnosed in the sixth to eight decade of life, and females are more commonly affected than men.^8^, ^9^, ^10^, ^11^ Diagnosis is challenging because of the indolent nature and similar clinical presentations to other obstructive biliary lesions including cholangiocarcinoma or choledocholithiasis. Treatment depends on grade and stage of the tumor; local tumors can be resected to attempt cure. Well-differentiated tumors are often treated with somatostatin analogs or peptide receptor radionuclide therapy, including lutetium-DotaTATE. Poorly differentiated or rapidly progressive tumors often require chemotherapy or targeted therapy, such as everolimus.^10^^,^^11^ Poorly differentiated gastrointestinal NETs typically have a poor prognosis. Reported median survival was 11 months in patients receiving palliative chemotherapy versus 1 month with best supportive care.^11^^,^^12^

The diagnosis of this rare biliary malignancy was achieved with minimal morbidity, enabling subsequent targeted oncologic therapy. This case underscores the value of adapting endoscopic tools to complex postsurgical anatomy, the diagnostic yield of cholangioscopy, and the importance of considering rare pathologies in biliary strictures.

## Patient consent

Written informed consent was obtained from the patient for publication of this case report and any accompanying images.

## Disclosure

All authors disclosed no financial relationships.

## Supplementary data

Video 1 The endoscopic diagnosis and management of a biliary neuroendocrine tumor is complicated by previous hepatobiliary surgery including Roux en Y choledochojejunostomy.

Video 2 The stricture caused by the biliary neuroendocrine tumor was treated with an uncovered metal stent.
